# Accelerated demand for interpersonal skills in the Australian post-pandemic labour market

**DOI:** 10.1038/s41562-023-01788-2

**Published:** 2024-01-08

**Authors:** David Evans, Claire Mason, Haohui Chen, Andrew Reeson

**Affiliations:** 1 Commonwealth Scientific and Industrial Research Organisation (CSIRO), Herston, Queensland Australia; 2 CSIRO, Herston, Queensland Australia; 3 CSIRO, Clayton, Victoria Australia; 4 CSIRO, Acton, Australian Capital Territory Australia

**Keywords:** Economics, Business and management, Economics

## Abstract

The COVID-19 pandemic has led to a widespread shift to remote work, reducing the level of face-to-face interaction between workers and changing their modes and patterns of communication. This study tests whether this transformation in production processes has been associated with disruptions in the longstanding labour market trend of increasing demand for interpersonal skills. To address this question, we integrate a skills taxonomy with the text of over 12 million Australian job postings to measure skills demand trends at the aggregate and occupational levels. We find that since the start of the pandemic, there has been an acceleration in the aggregate demand for interpersonal skills. We also find a strong positive association between an occupation’s propensity for remote work and the acceleration in interpersonal skills demand for the occupation. Our findings suggest that interpersonal skills continue to grow in importance for employment in the post-pandemic, remote work friendly labour market.

## Abstract (web summary)

The authors analyse over 12 million Australian job postings and find that since the start of the COVID-19 pandemic, there has been an acceleration in the aggregate demand for interpersonal skills.

## Main

Greater alignment between workers’ skills and employers’ skill requirements has several benefits, including increased job satisfaction for workers^1,2^, reduced labour turnover^1^ and higher employment and output^3^. Over time, shifts in firms’ production processes alter the tasks involved in production, leading to changes in the skills that employers demand of workers^4,5^. This process can cause misalignment between the skills that workers possess and the skills that employers require for production.

In recent decades, shifts in production processes have substantially increased employers’ demand for interpersonal skills^6–10^. This increase in demand is reflected in the growing employment shares of occupations with high interpersonal skill requirements (the between-occupation effect)^6–8^, along with increases in interpersonal skill requirements within occupations (the within-occupation effect)^9,10^. The between-occupation effect is largely due to the continued growth of services sector employment. The within-occupation effect can be attributed to the complementarity between interpersonal skills and new technologies^11,12^ and the growing importance of team production in many occupations^13^. The increasing importance of interpersonal skills for production has led to suggestions that education systems should have a greater focus on developing these skills to improve the alignment between workers’ skills and firms’ skill requirements^14–18^.

The COVID-19 pandemic required many firms to shift to remote modes of production, and with the threat of the pandemic reduced, many firms have maintained remote work arrangements^19–21^. It is unclear whether this widespread shift to remote work has disrupted the longstanding labour market trend of increasing demand for interpersonal skills. On the one hand, there is evidence that the shift to remote work substantially reduces the level of interaction between co-workers^22^ and causes workers to work in a more isolated manner^23^. There is also evidence that shifting to remote work causes workers to substitute synchronous communication for asynchronous communication and leads to sparser and more static collaboration networks within firms^21^. These findings suggest that perhaps interpersonal skills have become less important for production in the post-pandemic labour market. On the other hand, there is evidence that team cohesion and information brokers play important roles in preventing the negative effects of remote work on team performance^24^, suggesting that skills in working and communicating with others have become more valuable since the pandemic. In line with this suggestion, econometric analysis has shown that firms in regions of the United States that had longer stay-at-home orders during the COVID-19 pandemic increased their demand for communication skills by more than firms in regions that had shorter stay-at-home orders^25^. Similarly, anecdotal evidence from employers suggests that the demand for interpersonal skills has remained high since the pandemic^26–28^.

This study explores post-pandemic shifts in skills demand within occupations and at the aggregate level. Our primary analysis focuses on whether the shift to remote work has been associated with disruptions to the longstanding labour market trend of increasing demand for interpersonal skills. Our secondary analysis focuses on whether the shift to remote modes of production has been associated with changes in pre-existing demand trends for other major skill classes. Specifically, we explore whether the shift to delivering goods and services remotely has been associated with increasing demand for digital skills (such as the ability to create digital content and to communicate and collaborate using digital channels) and whether the demand for two other major skill classes (analytical and manual skills) has shifted in the post-pandemic period.

Our empirical strategy involves comparing the actual level of demand for each skill class in the post-pandemic period to the corresponding predicted level on the basis of pre-pandemic demand trends. Implementing this strategy involves several steps. First, we use the ESCO skills taxonomy to define our four high-level skill classes: interpersonal, analytical, digital and manual skills (see Table 1 for our mapping of ESCO skills to skill classes)^29^. Second, we integrate this taxonomy with the text of over 12 million Australian job postings to identify the skill classes mentioned in each new posting between 2015 and 2022. Third, we fit time-series models to these skills demand data in the pre-pandemic period and use the models to predict skills demand in the post-pandemic period. Fourth, we compare the actual demand trajectory for each skill class in the post-pandemic period to its predicted trajectory to measure the level of acceleration or deceleration in demand for the skill class. We perform this analysis at the occupational and aggregate levels.

**Table 1:** Level 2 ESCO skill groups represented within each skill class

Skill class	ESCO skill groups
Interpersonal	Counselling; providing information and support to the public and clients; advising and consulting; liaising and networking; negotiating; obtaining information verbally; presenting information; promoting, selling and purchasing; teaching and training; working with others; building and developing teams; leading and motivating; supervising people
Analytical	Analysing and evaluating information and data; calculating and estimating; conducting studies, investigations and examinations; documenting and recording information; managing information; information skills; monitoring developments in area of expertise; monitoring, inspecting and testing; measuring physical properties; processing information; developing objectives and strategies; performing administrative duties; making decisions
Digital	Accessing and analysing digital data; programming computer systems; setting up and protecting computer systems; using digital tools for collaboration, content creation and problem solving; using digital tools to control machinery; working with computers
Manual	Building and repairing structures; constructing; finishing interior or exterior of structures; installing interior or exterior infrastructure; assembling and fabricating products; cleaning; handling and disposing of waste and hazardous materials; handling and moving; handling animals; making moulds, casts, models and patterns; moving and lifting; positioning materials, tools or equipment; sorting and packaging goods and materials; tending plants and crops; transforming and blending materials; using hand tools; washing and maintaining textiles and clothing; driving vehicles; installing, maintaining and repairing electrical, electronic and precision equipment; installing, maintaining and repairing mechanical equipment; operating aircraft; operating machinery for the extraction and processing of raw materials; operating machinery for the manufacture of products; operating mobile plant; operating watercraft; using precision instrumentation and equipment; working with machinery and specialized equipment

We make several findings that support the hypothesis that interpersonal skills continue to grow in importance for employment in the post-pandemic, remote work friendly labour market. At the aggregate level, we find that the longstanding trend of increasing demand for interpersonal skills has accelerated in the post-pandemic period. We also find that this acceleration has primarily been driven by accelerated demand for communication and collaboration skills. At the occupational level, we find a strong positive association between an occupation’s propensity for remote work and the level of acceleration in interpersonal skills demand for the occupation, suggesting that these skills are increasingly important for remote workers. Our occupation-level analysis also reveals the individual occupations where the demand for interpersonal skills has accelerated or decelerated.

We also provide insights into the post-pandemic demand for other skill classes. In line with the notion that digital skills are increasingly important under remote modes of production, we find that the demand for these skills accelerated dramatically at the start of the pandemic and has since remained above its predicted trajectory. In terms of the other major skill classes, we find that the demand for analytical skills has largely continued along its pre-pandemic growth trajectory (albeit with a high degree of heterogeneity across occupations), and that the demand for manual skills has sharply decelerated in several occupations (albeit this deceleration is potentially due to coincident macroeconomic conditions).

## Results

### Aggregate skills demand

**Fig. 1:**
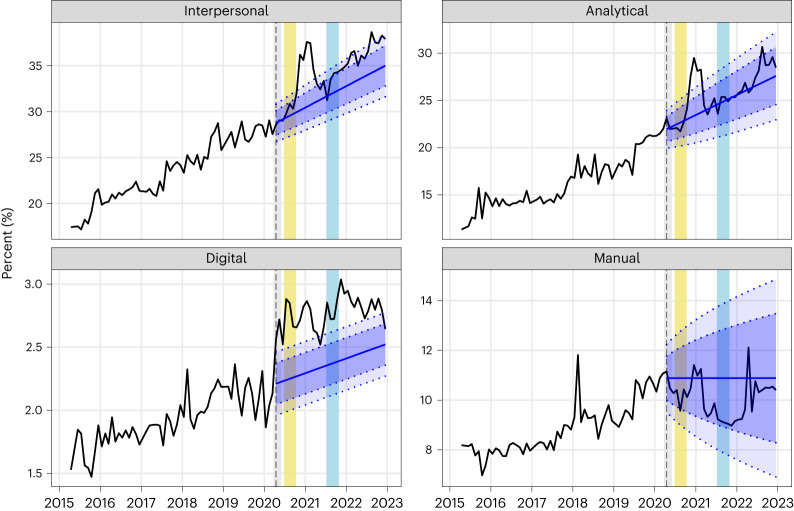
Skills demand in Australia: actual and predicted. The black line indicates the actual values for each skill between March 2015 and December 2022. Blue indicates the predicted values of ${\widetilde{{p}}}_{{jt}}$ in the post-pandemic period (April 2020 onwards): the central line is the mean predicted value, the inner dotted lines (bounding the dark blue region) indicate the 80% prediction interval and the outer dotted lines (bounding the dark and light blue regions) indicate the 95% prediction interval. The vertical dashed line indicates the first month of the post-pandemic period in Australia (April 2020). The coloured vertical bars indicate pandemic lockdown periods: grey shows the initial national lockdown (23 March 2020 to 31 May 2020), yellow shows the lockdown of Australia’s second most populous state of Victoria (8 July 2020 to 27 October 2020) and light blue shows the lockdown of Australia’s most populous state of New South Wales (26 June 2021 to 11 October 2021)^54^. Source data

The observed growth in demand for the interpersonal, digital, analytical and manual skill classes between March 2015 and December 2022 is visualized in Fig. 1. This figure shows the population-weighted proportion of job postings in each month *t* that mention the *j*th skill class ${\widetilde{p}}_{{jt}}$ (black line). This quantity is simply the raw proportion of postings mentioning the *j*th skill class reweighted to account for the number of workers in each occupation (see Methods for details). The figure shows that all skills classes have experienced increased demand over time, with interpersonal and analytical skills mentioned in a higher proportion of job postings than manual and digital skills.

Figure 1 compares ${\widetilde{p}}_{{jt}}$ for each skill class (black line) in the post-pandemic period to the corresponding predicted values (blue line) and prediction intervals (blue shading) based on a time-series model fitted to the pre-pandemic ${\widetilde{p}}_{{jt}}$ (see Table 2 for the parameter estimates). The figure shows that the pre-existing growth trend for interpersonal skills accelerated at the start of the pandemic, with ${\widetilde{p}}_{{jt}}$ exceeding the predicted trajectory for most of the post-pandemic period. The value of ${\widetilde{p}}_{{jt}}$ exceeded the upper bound of the 80% prediction interval for most of 2022 and fluctuated around the upper bound of the 95% prediction interval in the second half of 2022. To test whether this acceleration in demand is statistically significant, we compare the population-weighted proportion of postings that mention each skill class ${\bar{p}}_{j}$ in the last six months of the time series (July–December 2022) to the corresponding predicted value and 95% prediction interval (see Methods for details). We find that the ${\bar{p}}_{j}$ for interpersonal skills (37.7%) exceeds the upper bound of the 95% prediction interval (31.7–37.4%), indicating a statistically significant acceleration in demand.

**Table 2:** Estimated parameters of the state space models for the ${\widetilde{{p}}}_{{jt}}$ for each skill class *j*

	Interpersonal	Analytical	Digital	Manual
***α***	0.234	0.368	<0.001	0.482
***β***	<0.001	<0.001	<0.001	–
***l***_**0**_	0.174	0.118	0.0165	0.081
***b***_**0**_	0.002	0.002	<0.001	–
***σ***	0.010	0.010	0.001	0.064
RMSE (percentage point)	1.00	0.98	0.12	0.57

Demand for digital skills also accelerated with the onset of the pandemic and has since remained above the predicted level (although it has not grown materially throughout the post-pandemic period). As with interpersonal skills, the ${\bar{p}}_{j}$ for digital skills (2.8%) exceeds the upper bound of its 95% prediction interval (2.4–2.6%), indicating a statistically significant acceleration of demand. In contrast, the demand for manual skills has been below the predicted level for most of the post-pandemic period and the demand for analytical skills has closely followed the predicted trajectory for most of the post-pandemic period (with some evidence of an acceleration in demand in mid-2022). The ${\bar{p}}_{j}$ for manual (10.4%) and analytical (29.1%) skills fall well within their respective 95% prediction intervals (7.7–15.0% and 23.0–31.4%).

**Fig. 2:**
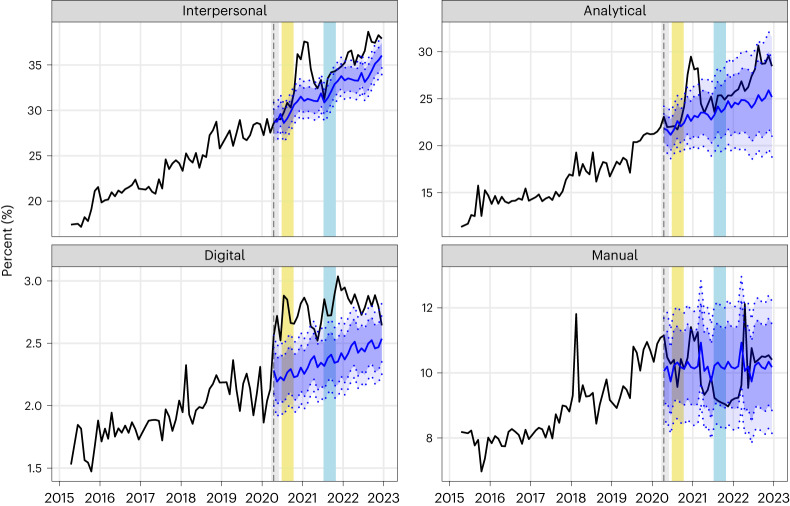
Skills demand in Australia: actual and predicted including seasonal factors. The black line indicates the actual values for each skill between March 2015 and December 2022. Blue indicates the predicted values of ${\widetilde{{p}}}_{{jt}}$ in the post-pandemic period (April 2020 onwards), based on models with seasonal components: the central line is the mean predicted value, the inner dotted lines (bounding the dark blue region) indicate the 80% prediction interval and the outer dotted lines (bounding the dark and light blue regions) indicate the 95% prediction interval. The vertical dashed line indicates the first month of the post-pandemic period in Australia (April 2020). The coloured vertical bars indicate pandemic lockdown periods: grey shows the initial national lockdown (23 March 2020 to 31 May 2020), yellow shows the lockdown of Australia’s second most populous state of Victoria (8 July 2020 to 27 October 2020) and light blue shows the lockdown of Australia’s most populous state of New South Wales (26 June 2021 to 11 October 2021)^54^. Source data

The results shown in Fig. 1 are robust to potential seasonality in skills demand. The predicted values and prediction intervals in Fig. 1 are based on time-series models fitted to the pre-pandemic data that maximize out-of-sample predictive accuracy (see Methods for details). These models do not contain seasonal components. To demonstrate the robustness of our results to potential seasonality, we fit time-series models with monthly seasonal components to the pre-pandemic data and use these models to generate predicted values and prediction intervals for the post-pandemic period. Figure 2 shows that these models lead to the same conclusions as the models without seasonal components.

**Fig. 3:**
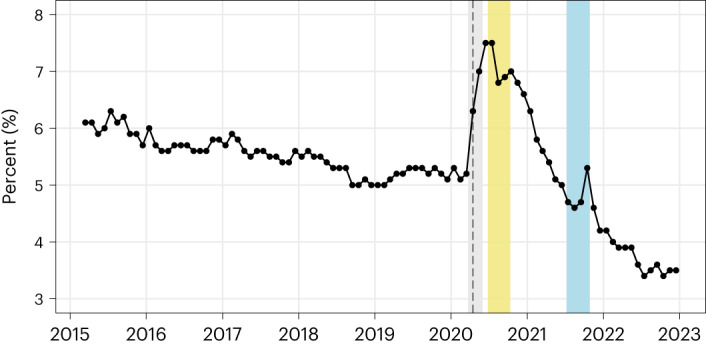
Australia’s seasonally adjusted unemployment rate across the period of our analysis (March 2015 to December 2022)^33^. The vertical dashed line indicates the first month of the post-pandemic period in Australia (April 2020). The coloured vertical bars indicate pandemic lockdown periods: grey shows the initial national lockdown (23 March 2020 to 31 May 2020), yellow shows the lockdown of Australia’s second most populous state of Victoria (8 July 2020 to 27 October 2020) and light blue shows the lockdown of Australia’s most populous state of New South Wales (26 June 2021 to 11 October 2021)^54^. Source data

Changes in macroeconomic conditions have probably driven some of the changes in skills demand shown in Fig. 1. Previous studies have shown that employers raise their skill requirements for new hires during periods of high unemployment (cyclical upskilling) and reduce these skill requirements as unemployment decreases (cyclical downskilling)^30–32^, suggesting that firms use slack labour markets as an opportunity to hire more skilled workers^30^. Changes in Australia’s unemployment rate over time (Fig. 3) enable the identification of periods in which cyclical upskilling and downskilling are likely to have occurred. Figure 3 shows a period of heightened unemployment in Australia from April 2020 to early 2021 following the onset of the pandemic^33^. Figure 1 shows that, as expected on the basis of previous studies, cyclical upskilling appears to have occurred during this period, with spikes in the demand for interpersonal and analytical (and to a lesser extent, manual) skills between late 2020 and early 2021. Figure 3 also shows a period of substantial labour market tightening between early 2021 and the end of 2022, with the unemployment rate decreasing to the historically low level of 3.5%. On the basis of previous studies, we expect cyclical downskilling to dampen the growth in skills demand during this period. Figure 1 shows that despite this expectation, the demand for interpersonal and digital skills has remained above expected levels. That is, the accelerated demand for these skills in the post-pandemic period has been robust to the expected dampening effect of cyclical downskilling.

Each of our skill classes comprises several detailed level 2 ESCO skills (see Table 1 for the full mapping). To identify the specific skills that have driven the shifts in aggregate demand for the skill classes, we compare the population-weighted proportion of postings that mention each level 2 ESCO skill *k* in July–December 2022 ${\bar{p}}_{k}$ to its predicted value ${\hat{p}}_{k}$ based on the pre-pandemic demand trajectory (see Methods for details). We then identify all skills where ${\bar{p}}_{k}$ differs materially from ${\hat{p}}_{k}$. We define this difference as material if (1) it exceeds 0.1 percentage point and (2) ${\bar{p}}_{k}$ falls outside its 80% prediction interval, suggesting that the difference is unlikely to be due to random variation in the time series.

**Fig. 4:**
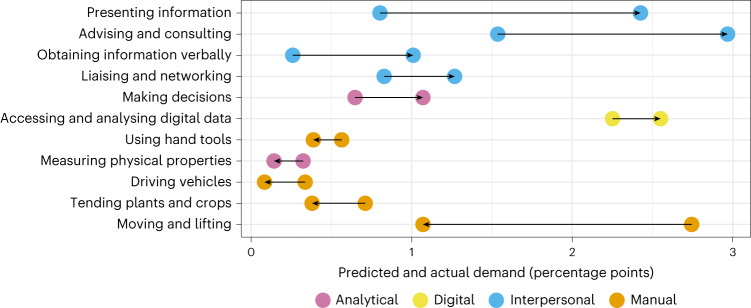
Level 2 ESCO skills where actual demand in July–December 2022 ${\bar{{p}}}_{{k}}$ differed materially from predicted demand ${\hat{{p}}}_{{k}}$ based on pre-pandemic trends. The start of the arrow indicates ${\hat{{p}}}_{{k}}$ and the end of the arrow indicates ${\bar{{p}}}_{{k}}$. Source data

Figure 4 shows the 11 skills where ${\bar{p}}_{k}$ differs materially from ${\hat{p}}_{k}$. The start of the arrow indicates the predicted value ${\hat{p}}_{k}$ and the end of the arrow indicates the actual value ${\bar{p}}_{k}$, so rightward arrows indicate accelerated demand and leftward arrows indicate decelerated demand. The skills are ordered by the level of acceleration ${{\bar{p}}_{k}-\hat{p}}_{k}$. The figure shows that the greatest accelerations in demand have occurred for four interpersonal skills related to communication and collaboration. The figure also shows that accelerated demand for skills in accessing and analysing digital data is the main driver of the accelerated demand for digital skills. Finally, the figure shows substantial deceleration in demand for four manual skills, potentially due to the cyclical downskilling described earlier.

### Remote work propensity and sustained effects on skills demand

If remote work arrangements increase the demand for interpersonal skills, then we expect occupation groups with higher propensities for remote work to have undergone greater acceleration in interpersonal skills demand than other occupation groups. To test whether this has occurred, we define the *i*th occupation group’s propensity for remote work *ρ*_*i*_ using three metrics**:** the proportion of workers in the occupation group that worked remotely in August 2021 according to Australian Census data (the census metric)^34^; the proportion of job postings for the occupation group in 2022 that offered remote work arrangements (the job postings metric); and the proportion of detailed lower-level occupations within the occupation group that are amenable to telework based on their task content^35^ (the job tasks metric). We also define the post-pandemic acceleration in demand for the *j*th skill class in the *i*th occupation group as

1 $${a}_{{ij}}={\bar{p}}_{{ij}}-{\hat{p}}_{{ij}}$$

where ${\bar{p}}_{{ij}}$ is the proportion of postings for the *i*th occupation that mentioned the *j*th skill class in July–December 2022 and ${\hat{p}}_{{ij}}$ is the corresponding predicted value based on the pre-pandemic trend (see Methods for details on how this quantity is estimated).

We then fit a simple linear regression model of the form

2 $${a}_{{ij}}={\beta}_{0}+{\beta}_{1}{\rho}_{i}+{\epsilon}_{{ij}}$$

to estimate the relationship between *ρ*_*i*_ and *a*_*ij*_ for each skill class *j*, where *β*_0_ and *β*_1_ are regression weights to be estimated and *ε*_*ij*_ is a zero-mean Gaussian error term. In fitting these models, we apply a robust weighting scheme (M-estimation with bisquare weighting) to limit the effect of outliers on the model and provide reliable estimates of the underlying relationship between *ρ*_*i*_ and *a*_*ij*_.

**Fig. 5:**
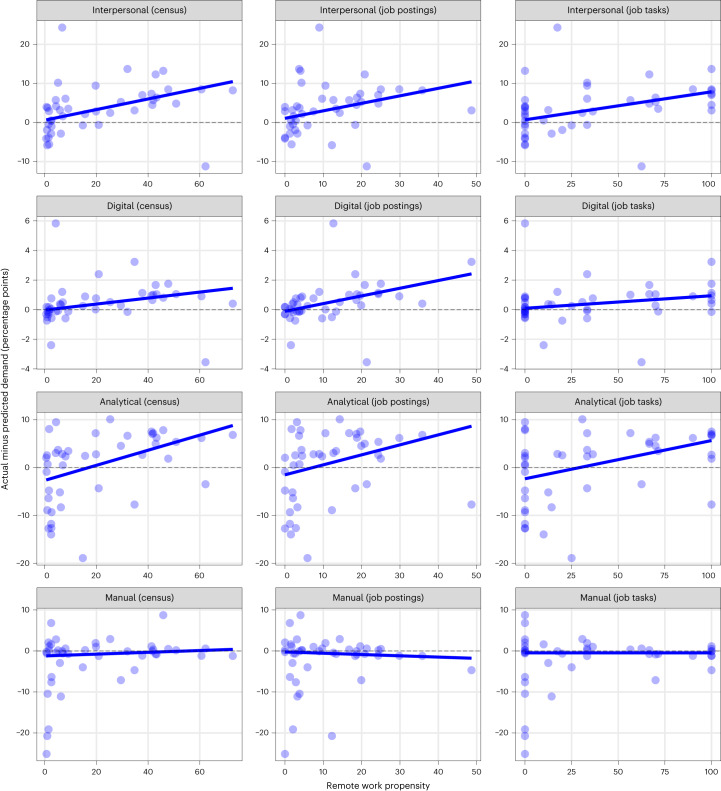
The values of *ρ*_*i*_ and *a*_*ij*_ for each level 2 ANZSCO occupation group *i*, skill class *j* and metric for *ρ*_*i*_ (census, job postings and job tasks metrics). Each line indicates the fitted values from a robust linear regression of *a*_*ij*_ on *ρ*_*i*_. There are *n* = 43 occupation groups. Two-sided *t*-tests of the hypothesis that *β*_1_ ≠ 0 provide the following *P* values on 41 degrees of freedom: *P* = 0.00007 (census metric), *P* = 0.003 (job postings metric) and *P* = 0.0002 (job tasks metric) for interpersonal skills; *P* = 0.003 (census and job tasks metrics) and *P* = 0.028 (job postings metric) for analytical skills; *P* = 0.00008 (census metric), *P* = 0.000001 (job postings metric) and *P* = 0.005 (job tasks metric) for digital skills; and *P* = 0.192 (census metric), *P* = 0.779 (job postings metric) and *P* = 0.503 (job tasks metric) for manual skills. Source data

Our fitted models for interpersonal skills show positive and statistically significant associations between *ρ*_*i*_ and *a*_*ij*_ for our three metrics of *ρ*_*i*_. The estimates are ${\hat{\beta }}_{1}=0.14$ (95% confidence interval (CI): 0.08, 0.20) under the census metric, ${\hat{\beta }}_{1}=0.19$ (95% CI: 0.06, 0.32) under the job postings metric and ${\hat{\beta }}_{1}=0.07$ (95% CI: 0.03, 0.11) under the job tasks metric. The top panels of Fig. 5 show these modelled values against the observed *ρ*_*i*_ and *a*_*ij*_ for each occupation, illustrating the positive associations between *ρ*_*i*_ and *a*_*ij*_. These results show that there has been greater acceleration in demand for interpersonal skills in occupations with higher propensities for remote work.

Our fitted models for analytical and digital skills also show positive and statistically significant associations between *ρ*_*i*_ and *a*_*ij*_. For analytical skills, the estimates are ${\hat{\beta }}_{1}=0.16$ (95% CI: 0.05, 0.27) under the census metric, ${\hat{\beta }}_{1}=0.21$ (95% CI: 0.00, 0.42) under the job postings metric and ${\hat{\beta }}_{1}=0.08$ (95% CI: 0.02, 0.14) under the job tasks metric. The corresponding estimates for digital skills are ${\hat{\beta }}_{1}=0.02$ (95% CI: 0.01, 0.03), ${\hat{\beta }}_{1}=0.05$ (95% CI: 0.03, 0.07) and ${\hat{\beta }}_{1}=0.01$ (95% CI: 0.00, 0.01) for the census, job postings and job tasks metrics, respectively. The second and third rows of Fig. 5 illustrate these positive associations. These results suggest that, post-pandemic, the acceleration in the demand for interpersonal, analytical and digital skills tended to be greatest in occupations with high propensity for remote work. In comparison, occupations that are not amenable to remote work arrangements tended to follow growth trends that were in line with pre-pandemic demand trends.

### The complementarity between skills and remote work

To further explore the complementarity between each skill class and remote work, we took a random sample of job postings posted in 2022 and tagged each posting with an indicator of whether it offered remote work (see Methods for details). We then tested whether the offer of remote work was positively associated with the mention of each skill class, controlling for occupation fixed effects. Specifically, we fitted a logistic regression model of the form

3 $$\log \left(\frac{{\rm{\pi} }_{{ij}}}{1-{\rm{\pi} }_{{ij}}}\right)={\beta }_{0}+{\beta }_{1}{r}_{i}+\mathop{\sum }\limits_{k=1}^{43}{\gamma }_{k}{d}_{{ik}}+{\epsilon }_{{ij}}$$

to the sample of job postings, where π_*ij*_ is the probability that the *i*th posting mentions the *j*th skill class, *r*_*i*_ takes the value 1 if the *i*th posting offers remote work and 0 otherwise and *d*_*ik*_ takes the value 1 if the *i*th posting is for occupation group *k* and 0 otherwise.

The fitted models suggest a high degree of complementarity between remote work and interpersonal and digital skills after controlling for occupation fixed effects. We estimate that job postings offering remote work are 1.20 times more likely (95% CI: 1.02–1.41) to mention interpersonal skills than postings not offering remote work. Similarly, we estimate that job postings offering remote work are 1.27 times more likely (95% CI: 1.05–1.53) to mention digital skills than postings not offering remote work. We find no statistically significant associations between the offer of remote work and mentions of the analytical or manual skill classes.

### Occupation-level skills demand

We also sought to shed light on occupation-level shifts in demand for skills classes in the post-pandemic labour market. To provide this insight, we compare each ${\bar{p}}_{{ij}}$ to its predicted value ${\hat{p}}_{{ij}}$ and prediction interval. If ${\bar{p}}_{{ij}}$ exceeds the upper bound of the 80% prediction interval, we conclude that the demand for the *j*th skill in the *i*th occupation has accelerated in the post-pandemic period (and vice versa). If ${\bar{p}}_{{ij}}$ falls within the 80% prediction interval, there is a reasonably high chance that any observed acceleration/deceleration is due to random variation in the time series, so we conclude that the demand for the *j*th skill in the *i*th occupation has continued along its pre-pandemic trajectory. Using the metric ${\bar{p}}_{{ij}}$ allows us to visualize patterns of acceleration/deceleration in skills demand across occupations without having to inspect all 172 time series (4 skill classes by 43 occupation groups), which would make it difficult to interpret overall patterns.

**Fig. 6:**
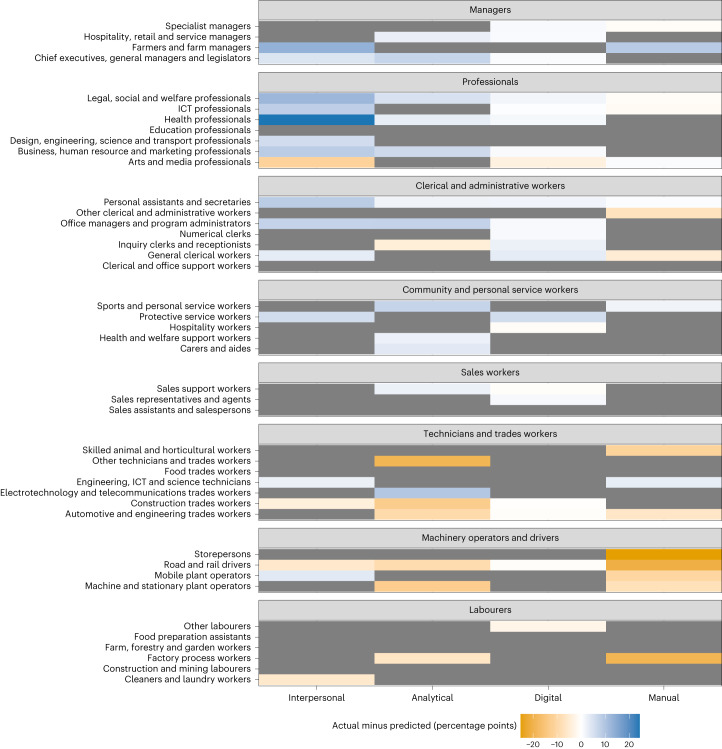
The level of acceleration/deceleration in the demand for each skill class within each occupation (level 2 ANZSCO) *a*_*ij*_ in the post-pandemic period. Occupations are grouped into eight higher-level occupation classes (level 1 ANZSCO). Cells are shaded in grey if ${\bar{{p}}}_{{ij}}$ falls within its 80% prediction interval. Source data

Figure 6 shows the level of acceleration/deceleration in the demand for each skill class in each occupation group ${a}_{{ij}}={\bar{p}}_{{ij}}-\,{\hat{p}}_{{ij}}$. Cells are shaded in grey if ${\bar{p}}_{{ij}}$ falls within its 80% prediction interval, indicating that any observed changes in skills trends can be attributed to random variation. Greater acceleration of demand for a skill class is denoted by more blue shading and greater deceleration of demand is denoted by more orange shading. The figure shows that the demand for interpersonal skills has accelerated in 13 occupations, continued along its pre-pandemic trajectory in 26 occupations and decelerated in only 4 occupations. The figure also shows that the acceleration in demand for interpersonal skills has been concentrated in managerial, professional and clerical and administrative occupations. This acceleration has been greatest for health professionals, where post-pandemic demand for several (level 2 ESCO) interpersonal skills (working with others, leading and motivating, presenting information and teaching and training) has exceeded expected levels based on pre-pandemic trends, suggesting that post-pandemic employment in the health sector requires increased levels of a range of interpersonal skills. The figure shows similar patterns in the acceleration/deceleration of demand for analytical and digital skills, although analytical skills have decelerated in a greater number of occupations (7) and the levels of acceleration in demand for digital skills tend to be relatively small across the occupations. Finally, the figure shows that the demand for manual skills has sharply decelerated for several classes of machinery operators and drivers, potentially due to cyclical downskilling.

## Discussion

This study reveals sustained disruptions to skills demand trends associated with the COVID-19 pandemic. Since the start of the pandemic, there has been an acceleration of the pre-existing trend of increasing demand for interpersonal skills. This acceleration has been driven by accelerated demand for communication and collaboration skills (for example, presenting information, advising and consulting). Furthermore, the accelerated demand for interpersonal skills is strongest in occupations that are amenable to remote working, suggesting that the increased reliance on digital channels for production has only served to reinforce the importance of interpersonal skills. These skills are perhaps required to overcome some of the negative effects of remote work, such as the thinning out and ossification of collaboration networks in firms^21^.

Post-pandemic disruptions to the demand for the other skill classes are mixed. Demand for digital skills has undergone a substantial acceleration at the aggregate level and within many occupations. Demand for analytical skills has continued along its pre-existing trajectory at the aggregate level, with several occupations undergoing acceleration and other occupations undergoing deceleration. Demand for manual skills has decelerated at the aggregate level, with sharp deceleration observed in several occupations, potentially due to cyclical downskilling.

The accelerated demand for interpersonal skills since the arrival of the pandemic has occurred despite an extended period of labour market tightening. Previous research has shown that tighter labour markets cause employers to reduce their skill demands of workers^32^. As such, we expect the tightening of the Australian labour market between March 2020 and December 2022, where the unemployment rate decreased from 5.3% to 3.5%^33^, to dampen the growth in demand for interpersonal (and other) skills. Despite this expectation, we observe an acceleration of demand for interpersonal skills at the aggregate level and within several occupations, and deceleration within very few occupations. Such a finding is not without precedent, with similar structural increases in skills demand observed during a period of labour market tightening in the years following the Great Recession^36^.

Our analysis complements previous findings on the increasing demand for interpersonal skills before the pandemic^6–10^. We show that this trend has accelerated at the aggregate level and has persisted or accelerated in almost all occupation groups. As such, our analysis supports the ongoing relevance of previous policy recommendations that education systems should have a greater focus on developing the interpersonal skills of students and workers^14–18^.

A limitation of this research is that it relies on the skills described in job postings to determine what skills are becoming more sought after in the labour market. Job postings do not provide a comprehensive list of all the skills needed to perform a given role^37^. However, since job postings serve as a means through which employers communicate the skills they value in the role, they are commonly used to understand changing skills trends^31,38–40^. They also provide more up-to-date and localized information about skills trends than occupational surveys such as O*NET^41^.

We provide three potential extensions of this research. First, our method could be extended to monitor the demand for detailed skill classes within detailed occupation classes (as opposed to the broad skill and occupation classes we have used in this study). This information could help labour market participants identify emerging skills and skills in decline in different segments of the labour market. Monitoring skills demand at these detailed levels would require use of natural language processing algorithms (rather than exact match) to improve the detection of skills so that enough skills data can be captured at the more detailed occupation level for reliable monitoring.

Second, Fig. 6 suggests a potential post-pandemic divergence in the skills required for ‘high skill’ and ‘low skill’ occupations. Based on Australia’s standard occupation taxonomy^42^ and the number of skills mentioned in job postings, skill requirements are greatest for professionals and managers, and smallest for labourers, and machinery operators and drivers (with the other occupation groups somewhere in between). Figure 6 shows that skills demand has generally accelerated for professionals and managers, and decelerated for labourers, and machinery operators and drivers, suggesting increasing polarization of workplace skills across occupations. This type of skills polarization constrains the transitions that workers can make between occupations and can cause low skill workers to become stuck in low skill and low wage occupations^43^. A more detailed analysis of skills polarization could reveal changes in the level of polarization over time and inform policy responses to the negative effects of polarization.

Finally, data on how the supply of skills is changing over time in different parts of the labour force could be combined with our data on the changing demand for skills to identify emerging skill mismatches. This integrated analysis would further inform skills investments and decisions being made by individuals and policymakers.

## Methods

### Defining skills

We used the ESCO skills hierarchy to define the set of skill classes for our analysis^29^. This hierarchy groups over 13,000 detailed job-related skills into a smaller number of broader skill groups^44,45^. We mapped these broader skill groups to our four skill classes of interest, as shown in Table 1.

### Adzuna Australia job postings data

This research was based on job postings data provided by Adzuna Australia. Adzuna Australia is an aggregator of job postings that has been operating in Australia since 2013. Adzuna Australia’s database contains job postings from a variety of sources: postings that employers and recruitment agencies post on Adzuna Australia’s online platform, postings listed in one of Australia’s largest newspapers and postings that Adzuna Australia scrapes from other websites (for example, employers’ websites)^46^. This database’s coverage of Australian job postings closely matches the coverage of the Lightcast (formerly Burning Glass) database, which contains the near-universe of job postings^46^.

Adzuna Australia’s scraping process increases the database’s coverage of the population of job postings but also increases the likelihood of duplicate postings entering the database^47^. To address this problem, Adzuna Australia screens the postings it scrapes from other sources and removes suspected duplicates^46^. We applied an additional filter to remove other suspected duplicates (postings with the same job title, same location, similar posting dates and near-identical job descriptions)^47^.

Each job posting in the Adzuna Australia database was tagged with the job’s occupation title (as described by the poster) and the date when the job was posted (or scraped) to Adzuna Australia’s online platform. A natural language processing-based algorithm was used to match each posting’s occupation title and role description to an occupation in the Australian and New Zealand Standard Classification of Occupations (ANZSCO; Australia’s standard occupation taxonomy)^42^.

The data set used for our analysis contained all 12,471,217 postings that entered the Adzuna Australia database between March 2015 and December 2022. The monthly count of postings (sample size) varied from 44,137 (March 2020) to 339,306 (November 2022). The total count of postings mentioning each skill class is 3,467,292 for interpersonal skills, 2,384,950 for analytical skills, 292,396 for digital skills and 905,295 for manual skills.

### Using job postings data to measure skills demand

We used an exact match algorithm to identify the detailed ESCO skills mentioned in each job posting in the database. This algorithm uses the English language preferred and alternative labels for each of the 13,890 skills listed in the ESCO skills pillar^44^. When a job posting contained one of the preferred or alternative skill labels, it was tagged as requiring the relevant skill. The hierarchical structure of the ESCO taxonomy was then used to aggregate these skills matches to one of the ESCO level 2 skill groups, which we then assigned to one of our four skills classes (Table 1). While the use of an exact match algorithm means that skill classes will go undetected whenever there is a close-but-inexact match, such an approach is commonly used for monitoring changes in the demand for different skill classes over time.

We used these data to compute the proportion of new postings for each occupation *i* in each month *t* that mention each skill class *j* (*p*_*ijt*_) and level 2 ESCO skill *k* (*p*_*kjt*_). These metrics formed the basis of the analysis in this study.

To measure the aggregate demand for each skill class, we developed a metric that captures both within-occupation and between-occupation changes in demand for the skill over time. Here we computed the population-weighted proportion of all job postings mentioning each skill as

4 $${\widetilde{p}}_{{jt}}=\sum _{i}{\alpha }_{{it}}{p}_{{ijt}}$$

where each *p*_*ijt*_ was weighted by occupation group *i*’s (level 2 ANZSCO) share of total workers in month *t* (*α*_*it*_) based on official labour statistics^33^. This weighting method corrects for the overrepresentation of some occupations (and underrepresentation of others) in the job postings data, which can cause the (unweighted) proportion of postings mentioning each skill to be a misleading measure of the aggregate demand for that skill^31,48–50^. The metric ${\widetilde{p}}_{{jt}}$ reflects both within-occupation changes in skills demand via changes in *p*_*ijt*_ and between-occupation changes in skills demand via changes in *α*_*it*_.

### Time-series models

#### Overview

Our method of measuring the post-pandemic acceleration/deceleration in demand for the *j*th skill class (or *k*th level 2 ESCO skill) at both the aggregate and occupational levels involves three steps:

1. Fitting a state space exponential smoothing model to the time-series data on the demand for the skill class in the pre-pandemic period.
2. Using the fitted model to generate predictions and prediction intervals of the demand for the skill class in the post-pandemic period.
3. Comparing observed demand to predicted demand in the post-pandemic period.

#### Model selection and estimation

In step 1 of this method, we used the ets function in R’s forecast package to select the best model from the class of ‘innovations’ (single source of error) state space exponential smoothing models^51^. We used this class of models as the models within it are flexible enough to fit a range of economic time-series data and provide accurate predictions of future observations relative to alternative models^52^.

The ets function fits models with different combinations of additive and multiplicative trend, seasonal and error components, and selects the model that minimizes the Akaike information criterion (AIC):

5 $${\rm{AIC}}=2k-\mathrm{ln}({\hat{L}}).$$

Here, *k* is the number of model parameters estimated and $\hat{L}$ is the maximized value of the model’s likelihood function. That is, the ets function selects the model that maximizes the likelihood of the data subject to a penalty for the model’s complexity. Imposing this penalty reduces the likelihood of selecting models that overfit the training data and typically leads to the selection of models with better out-of-sample predictive accuracy^51^.

For the ${\widetilde{p}}_{{jt}}$ for interpersonal, analytical and digital skills, the above process led to the selection of models with additive error and trend components and no seasonal component. The state space equations of this model are given by

6 $${\widetilde{p}}_{{jt}}={l}_{t-1}+{b}_{t-1}+{\epsilon }_{t}$$

7 $${l}_{t}={l}_{t-1}+{b}_{t-1}+\alpha {\epsilon }_{t}$$

8 $${b}_{t}={b}_{t-1}+\beta {\epsilon }_{t}$$

where *l*_*t*_ is the time-series level at time *t*, *b*_*t*_ is the time-series slope at time *t*, ${\epsilon }_{t}={y}_{t}-{l}_{t-1}-{b}_{t-1} \sim {\rm{NID}}(0,{\sigma }^{2})$ is the error term (where NID denotes normally and independently distributed) and *α* and *β* are constants to be estimated. Note that we have omitted the subscript *j* from *l*, *b* and *ϵ* for ease of expression.

For the ${\widetilde{p}}_{{jt}}$ for manual skills, the above process led to the selection of a model with multiplicative errors and no trend or seasonal component. The state space equations of this model are given by

9 $${\widetilde{p}}_{{jt}}={l}_{t-1}(1+{\epsilon }_{t})$$

10 $${l}_{t}={l}_{t-1}(1+\alpha {\epsilon }_{t})$$

where ${\epsilon }_{t} \sim {\rm{NID}}(0,{\sigma }^{2})$. Again, we have omitted the subscript *j* from *l* and *ϵ*.

Table 1 shows the parameter estimates for each of these state space models trained on data from the pre-pandemic period (March 2015 to March 2020). The table also provides each model’s root mean square error (RMSE) on the training data.

#### Prediction intervals and inference

To generate predictions and prediction intervals of the demand for each skill class in the post-pandemic period (step 2 of the above method), we used the fitted model to simulate sample paths of ${\widetilde{p}}_{{jt}}$ (for aggregate demand) or *p*_*ijt*_ (for occupation-level demand) from April 2020 to December 2022. Then, in each month of the post-pandemic period, we computed the mean of these sample paths as the predicted value and the (*k*/2)th and (1 − *k*/2)th percentiles of these sample paths as the lower and upper bounds, respectively, of the *k*% prediction interval. These prediction intervals provided an indication of the range of trajectories each time series could have taken if it had continued to follow its pre-pandemic stochastic process. Comparing these trajectories to the actual values allowed us to infer whether there was an acceleration or deceleration in demand.

We used a similar simulation approach to generate prediction intervals for ${\bar{p}}_{{ij}}$ (the mean proportion of postings in the *i*th occupation that mention the *j*th skill class in the last six months of 2022) and ${\bar{p}}_{j}$ (the population-weighted mean proportion of postings that mention the *j*th skill class in the last six months of 2022). This simulation approach is described in ref. ^53^.

#### Robustness of results to the inclusion of seasonal factors

As noted above, our model selection process led to the selection of models with no seasonal component. To test the robustness of our results to seasonal factors, we took the selected models and added additive seasonal components. The resulting state space equations of the model for interpersonal, analytical and digital skills are given by

11 $${\widetilde{p}}_{{jt}}={l}_{t-1}+{b}_{t-1}+{s}_{t-m}+{\epsilon }_{t}$$

12 $${l}_{t}={l}_{t-1}+{b}_{t-1}+\alpha {\epsilon }_{t}$$

13 $${b}_{t}={b}_{t-1}+\beta {\epsilon }_{t}$$

14 $${s}_{t}={s}_{t-m}+\gamma {\epsilon }_{t}$$

where *s*_*t*_ is the seasonal component at time *t*, and *m* is the number of seasons per year. We set *m* = 12 to account for seasonal variation at the monthly level.

The resulting state space equations of the model for manual skills are given by

15 $${\widetilde{p}}_{{jt}}=(l_{t-1}+{s}_{t-m})(1+{\epsilon }_{t})$$

16 $${l}_{t}={l}_{t-1}+\alpha \left({l}_{t-1}+{s}_{t-m}\right){\epsilon }_{t}$$

17 $${s}_{t}={s}_{t-m}+\gamma \left({l}_{t-1}+{s}_{t-m}\right){\epsilon }_{t}$$

Figure 2 is a reproduction of Fig. 1 using the above models with seasonal components. It shows that these models led to the same conclusions as the models without seasonal components, with post-pandemic demand for interpersonal and digital skills exceeding the predicted levels.

### Identifying offers of remote work arrangements in job postings

We randomly sampled 6,800 job postings posted in 2022 and created an indicator variable for whether each posting offered remote work arrangements. We then used these data to analyse the complementarity between remote work and each skill class and computed the proportion of postings offering remote work arrangements in each occupation group.

To identify remote work arrangements in job postings, we utilized the capabilities of ChatGPT 3.5 via OpenAI’s API. Each job posting was presented to the model to determine whether the content suggested a ‘remote work arrangement’. To validate ChatGPT’s classifications, we engaged two human annotators to assess a random subset comprising 36 AI-evaluated job postings.

Out of these 36 postings, consensus with the human annotators was reached for 31, suggesting the task’s feasibility. ChatGPT’s categorizations closely mirrored human judgments, with discrepancies arising in only three postings when compared against each individual annotator. This considerable congruence indicates ChatGPT’s reliability and accuracy in identifying ‘remote work arrangements’ within job postings.

### Reporting summary

Further information on research design is available in the Nature Portfolio Reporting Summary linked to this article.

### Supplementary information

Reporting Summary

### Source data

Source Data Fig. 1 Statistical source data.

Source Data Fig. 2 Statistical source data.

Source Data Fig. 3 Statistical source data.

Source Data Fig. 4 Statistical source data.

Source Data Fig. 5 Statistical source data.

Source Data Fig. 6 Statistical source data.

## Data Availability

All relevant necessary data to reproduce the results in the paper are publicly available in the Dryad Digital Repository at 10.5061/dryad.sf7m0cgbx. Source data are provided with this paper.

## Code availability

The code required to transform the data into the statistics and outputs reported in the paper are publicly available at https://github.com/davide8484/skills-demand.
