# Supplementary material for: Accelerated demand for interpersonal skills in the Australian post-pandemic labour market
Source: Nat Hum Behav. 2024 Jan 8;8(1):32–42. doi: 10.1038/s41562-023-01788-2 (PMC10810758; doi:10.1038/s41562-023-01788-2)
Supplement: Supplementary file 1 — Reporting Summary [file 41562_2023_1788_MOESM1_ESM.pdf]

nature portfolio

Corresponding author(s): David Evans

Last updated by author(s): 6/11/2023

Reporting Summary

Nature Portfolio wishes to improve the reproducibility of the work that we publish. This form provides structure for consistency and transparency in reporting. For further information on Nature Portfolio policies, see our Editorial Policies and the Editorial Policy Checklist.

Statistics

For all statistical analyses, confirm that the following items are present in the figure legend, table legend, main text, or Methods section.

n/a	Confirmed	
☐	☒	The exact sample size (*n*) for each experimental group/condition, given as a discrete number and unit of measurement
☐	☒	A statement on whether measurements were taken from distinct samples or whether the same sample was measured repeatedly
☐	☒	The statistical test(s) used AND whether they are one- or two-sided<br>*Only common tests should be described solely by name; describe more complex techniques in the Methods section.*
☒	☐	A description of all covariates tested
☒	☐	A description of any assumptions or corrections, such as tests of normality and adjustment for multiple comparisons
☐	☒	A full description of the statistical parameters including central tendency (e.g. means) or other basic estimates (e.g. regression coefficient) AND variation (e.g. standard deviation) or associated estimates of uncertainty (e.g. confidence intervals)
☐	☒	For null hypothesis testing, the test statistic (e.g. *F*, *t*, *r*) with confidence intervals, effect sizes, degrees of freedom and *P* value noted<br>*Give P values as exact values whenever suitable.*
☒	☐	For Bayesian analysis, information on the choice of priors and Markov chain Monte Carlo settings
☒	☐	For hierarchical and complex designs, identification of the appropriate level for tests and full reporting of outcomes
☒	☐	Estimates of effect sizes (e.g. Cohen's *d*, Pearson's *r*), indicating how they were calculated

Our web collection on statistics for biologists contains articles on many of the points above.

Software and code

Policy information about availability of computer code

Data collection	We use the open source software R (version 4.2.0) to generate the data sets used for this study. The R packages used to generate these data sets are tidyverse and lubridate.
Data analysis	We use the open source software R (version 4.2.0) to perform all of the modelling and analysis in this study. The R packages used for the analysis are: tidyverse; scales; lubridate; forecast; MASS. A program that transforms the data above into the statistics and outputs in the manuscript is publicly available at https://github.com/davide8484/skills-demand.

For manuscripts utilizing custom algorithms or software that are central to the research but not yet described in published literature, software must be made available to editors and reviewers. We strongly encourage code deposition in a community repository (e.g. GitHub). See the Nature Portfolio guidelines for submitting code & software for further information.

Data

Policy information about availability of data

All manuscripts must include a data availability statement. This statement should provide the following information, where applicable:

- Accession codes, unique identifiers, or web links for publicly available datasets
- A description of any restrictions on data availability
- For clinical datasets or third party data, please ensure that the statement adheres to our policy

Adzuna Australia provided access to its proprietary job postings data for this study. We have integrated these data with the publicly available ANZSCO occupational

taxonomy and the ESCO skills taxonomy, enabling the measurement of skills demand in different occupations over time. While we are not permitted to share Adzuna Australia's raw job postings data publicly, we are able to share all the derived data used in our analysis (e.g., the monthly count of job postings, the proportion of job postings mentioning each skill class in each occupation etc.). These data sets are available at https://doi.org/doi:10.5061/dryad.sf7m0cgbx

All relevant necessary data to reproduce the results in the paper are publicly available in the Dryad Digital Repository: https://doi.org/doi:10.5061/dryad.sf7m0cgbx

Research involving human participants, their data, or biological material

Policy information about studies with human participants or human data. See also policy information about sex, gender (identity/presentation), and sexual orientation and race, ethnicity and racism.

Reporting on sex and gender	Our analysis uses job postings data, which are not broken down by sex or gender. Therefore, our results do not provide outputs by sex or gender.
Reporting on race, ethnicity, or other socially relevant groupings	Our analysis uses job postings data. We categorize these postings by occupation using Australia's standard occupation taxonomy. We use the ESCO skills taxonomy to identify the skills mentioned in each posting.<br><br>Our data contain no indications of race, ethnicity, or other socially relevant groupings, so our do not provide breakdowns by these variables.
Population characteristics	See above (our study is not based on human research participants).
Recruitment	Our analysis uses job postings data, so no study participants were recruited.
Ethics oversight	Commonwealth Scientific and Industrial Research Organisation

Note that full information on the approval of the study protocol must also be provided in the manuscript.

Field-specific reporting

Please select the one below that is the best fit for your research. If you are not sure, read the appropriate sections before making your selection.

☐ Life sciences ☒ Behavioural & social sciences ☐ Ecological, evolutionary & environmental sciences

For a reference copy of the document with all sections, see nature.com/documents/nr-reporting-summary-flat.pdf

Behavioural & social sciences study design

All studies must disclose on these points even when the disclosure is negative.

Study description	This study quantitatively analyses job postings data to measure trends in skills demand between 2015 and 2022 and identify shifts in skills demand that have occurred since the onset of the COVID-19 pandemic.
Research sample	Our sample contains the 12,471,217 job postings collected by the aggregator Adzuna Australia between March 2015 and December 2022. This database contains job postings from a variety of sources: postings that employers and recruitment agencies post on Adzuna Australia's online platform; postings listed in one of Australia's largest newspapers; and postings that Adzuna Australia scrapes from other websites. This database provides a large and representative sample of Australian job postings, closely matching the coverage of the Lightcast (formerly Burning Glass) database, which contains the near-universe of job postings.<br><br>Our rationale for choosing this study sample is to measure skills demand trends in the pre-pandemic period (2015-2020) and post-pandemic period (2020-2022) to detect changes in demand. The text of job postings indicates the skills employers demand, so analysing a large sample of Australian postings over time allows us to measure how the demand for different skills is changing over time in Australia.<br><br>Researchers were not blinded to the study hypothesis.
Sampling strategy	We analyse the entire population of job postings. No further sampling of postings was performed.
Data collection	We use job postings collected by the aggregator Adzuna Australia.
Timing	The job postings data set contains monthly data covering the period March 2015 to December 2022.
Data exclusions	No data were excluded from the analysis.
Non-participation	The data are job postings, so there were no individual participants.
Randomization	The data are job postings, so no randomization of participants etc. was required.

Reporting for specific materials, systems and methods

We require information from authors about some types of materials, experimental systems and methods used in many studies. Here, indicate whether each material, system or method listed is relevant to your study. If you are not sure if a list item applies to your research, read the appropriate section before selecting a response.

Materials & experimental systems

n/a	Involved in the study
☒	☐ Antibodies
☒	☐ Eukaryotic cell lines
☒	☐ Palaeontology and archaeology
☒	☐ Animals and other organisms
☒	☐ Clinical data
☒	☐ Dual use research of concern
☒	☐ Plants

Methods

n/a	Involved in the study
☒	☐ ChIP-seq
☒	☐ Flow cytometry
☒	☐ MRI-based neuroimaging
